# Nicotinamide-Rich Diet in DBA/2J Mice Preserves Retinal Ganglion Cell Metabolic Function as Assessed by PERG Adaptation to Flicker

**DOI:** 10.3390/nu12071910

**Published:** 2020-06-27

**Authors:** Tsung-Han Chou, Giovanni Luca Romano, Rosario Amato, Vittorio Porciatti

**Affiliations:** 1 Bascom Palmer Eye Institute, University of Miami, Miami, FL 33136, USA; giovanniluca.romano@unict.it (G.L.R.); rosario.amato@biologia.unipi.it (R.A.); vporciatti@med.miami.edu (V.P.); 2 Department of Biomedical and Biotechnological Sciences, University of Catania, CT 95124 Catania, Italy; 3 Department of Biology, University of Pisa, PI 56126 Pisa, Italy

**Keywords:** adaptation, retinal ganglion cell, pattern electroretinogram, nicotinamide, mitochondria

## Abstract

Flickering light increases metabolic demand in the inner retina. Flicker may exacerbate defective mitochondrial function in glaucoma, which will be reflected in the pattern electroretinogram (PERG), a sensitive test of retinal ganglion cell (RGC) function. We tested whether flicker altered the PERG of DBA/2J (D2) glaucomatous mice and whether vitamin B3-rich diet contributed to the flicker effect. D2 mice fed with either standard chow (control, *n* = 10) or chow/water enriched with nicotinamide (NAM, 2000 mg/kg per day) (treated, *n* = 10) were monitored from 3 to 12 months. The PERG was recorded with superimposed flicker (F-PERG) at either 101 Hz (baseline) or 11 Hz (test), and baseline-test amplitude difference (adaptation) evaluated. At endpoint, flat-mounted retinas were immunostained (RBPMS and mito-tracker). F-PERG adaptation was 41% in 3-month-old D2 and decreased with age more in control D2 than in NAM-fed D2 (GEE, *p* < 0.01). At the endpoint, F-PERG adaptation was 0% in control D2 and 17.5% in NAM-fed D2, together with higher RGC density (2.4×), larger RGC soma size (2×), and greater intensity of mitochondrial staining (3.75×). F-PERG adaptation may provide a non-invasive tool to assess RGC autoregulation in response to increased metabolic demand and test the effect of dietary/pharmacological treatments on optic nerve disorders.

## 1. Introduction

Glaucoma, a progressive optic neuropathy characterized by retinal ganglion cell (RGC) degeneration and visual field loss, is a leading cause of irreversible blindness worldwide [1]. Primary open-angle glaucoma (POAG) is the most frequent type of glaucoma at old age, an elevated intraocular pressure (IOP) being the main risk factor [2]. IOP lowering, however, does not always prevent disease progression due to the multifactorial nature of the glaucomatous disease. Therefore, neuroprotective strategies aiming at slowing down POAG progression have been developed in recent years to complement IOP lowering therapy [3,4,5].

The DBA/2J (D2) mouse is a widely used model of glaucoma, which recapitulates the hallmark features of the human disease [6,7]. An altered retinal ganglion cell (RGC) function in D2 glaucoma mouse model can be non-invasively detected by pattern electroretinogram (PERG) before alteration of RGC structure and RGC loss [8,9]. Recent studies in D2 mice reported that RGCs go through progressive stages of mitochondrial stress and metabolite depletion that occur prior to neurodegeneration, at a time-point that corresponds with early decrease in electrical activity as assessed by PERG [10,11]. These studies also reported that vitamin B3 (nicotinamide, NAM) treatment robustly prevents development of glaucoma in D2 mice. Other studies also reported that group B vitamins and other supplementation protect from inflammation-associated RGC degeneration in a mouse model of optic nerve injury [12,13].

As RGCs are extremely high-maintenance neurons under normal conditions [14,15], it is conceivable that the addition of flickering light to the visual stimulus—known to increase metabolic demand and cause rapid vasodilation [16,17]—will increase the likelihood of an energetic crisis in D2 mice prone to glaucoma. We recently developed a non-invasive tool for testing neurovascular autoregulatory dynamics under metabolic challenge in the mouse eye in vivo [18]. The tool consists of superimposing a flickering light at 11 Hz to the pattern stimulus for PERG recording, resulting in a flicker PERG (F-PERG) response. The F-PERG undergoes amplitude reduction over time (adaptation) associated with vasodilation. Here, we tested the hypothesis that F-PERG adaptation will be progressively reduced in D2 glaucoma, but it will be spared when D2 mice are fed with a vitamin B3-rich diet in association with sparing of RGCs and mitochondria.

## 2. Materials and Methods

### 2.1. Animals and Husbandry

In the present study, 20 DBA/2J (D2) mice 3 months old purchased from Jackson Labs (Bar Harbor, ME, USA) were used. All procedures were performed in compliance with the Association for Research in Vision and Ophthalmology (ARVO) statement for use of animals in ophthalmic and vision research. The experimental protocol was approved by the Animal Care and Use Committee of the University of Miami (Project protocol number: 16-247). All mice were maintained in a cyclic light environment (12 h light: 50 lux–12 h: dark) and fed with modification of nicotinamide enriched chow (TestDiet^®^, St. Louis, MO, USA, 1816036-204) with 6 g NAM in drinking water. The approximate intake dosage for each mouse was 2000 mg/kg NAM per day [10]. All procedures and testing were performed under anesthesia by means of intraperitoneal injections (0.5–0.7 mL/kg) of a mixture of ketamine (42.8 mg/mL) and xylazine (8.6 mg/mL).

### 2.2. Assessment of RGC Function and Adaptation of RGC Function during Flicker Light in D2 Mice

RGC function was assessed by Pattern Electroretinogram (PERG), an electrical signal that specifically depends on the presence of functional RGCs [19] and is commonly used in human and experimental models of glaucoma and optic neuropathies [10,20]. The PERG technique for simultaneous recording from both eyes has been previously described in detail [9,21]. In the present study, we used a commercially available instrument for PERG recording (Jorvec Corp., Miami, FL, USA) modified to superimpose a flickering field to the patterned stimulus [18]. In brief, anesthetized mice were gently restrained in a holder allowing unobstructed vision and kept at a constant body temperature of 37.0 °C using feedback-controlled heating pad controlled by a rectal probe. Pupils were undilated and small (<1 mm), which insured a large depth of focus. PERG signals were recorded from a subcutaneous stainless steel needle (Grass, West Warwick, RI, USA) placed in the snout. The reference and ground electrodes were similar needles placed medially on the back of the head and at the root of the tail, respectively. Visual stimuli were presented at each eye independently from 10 cm distance and consisted of contrast-reversal of gratings (0.05 cycles/deg, 98% contrast) generated on two light-emitting diode (LED) tablets (15 × 15 cm square field, 800 cd/m^2^ mean luminance) alternating at slightly different frequencies around 1 Hz (OD, 1.016 Hz; OS, 1.008 Hz). Independent PERG signals from each eye were retrieved using one channel continuous acquisition and phase-locking average over 372 epochs for each eye. PERG amplitude was measured peak-to-trough using a software that automatically detected the positive peak and the negative trough in the PERG waveform (typically the P1 peak to the N2 trough). Noise responses were obtained by computing the difference between even and odd epochs [22]. The patterned LED display was surrounded by a LED square frame (internal size 15 × 15 cm, external size 18 × 18 cm) flickering light. The flickering frame had the same mean luminance of the patterned field, and could be modulated (square-wave, 50% duty cycle) at either 101 Hz or 11 Hz at constant mean luminance. These frequencies were asynchronous with the pattern reversal frequencies. At 101 Hz, flickering light could not be perceived by human observers. The sequence of PERG recording with superimposed flicker (F-PERG) was F-PERG_101 Hz (baseline), F-PERG_11 Hz (test), and F-PERG_101 Hz (second baseline), and each PERG measurement being the average of 744 epochs from each eye. F-PERG adaptation was defined as the amplitude change upon transition from F-PERG baseline to the F-PERG test.

### 2.3. RGC and Mitochondria Immunohistochemistry Method

Three young D2 mice (3-month-old) with other six longitudinal D2 mice 13 months old (treated, *n* = 3; controls, *n* = 3) were euthanized and fixed by systemic perfusion with 4% *w*/*v* paraformaldehyde in phosphate buffer saline 0.1 M pH 7.4 (PBS). Hereafter, eyeballs were enucleated and immerged in the same fixing solution for two hours at room temperature. After the fixation process, eyes were stored at 4 °C in 30% *w*/*v* sucrose solution in PBS. Immediately before the immunostaining, each eyeball was dissected in order to separate the retina from the retinal pigmented epithelium, sclera, and components of the anterior eye tissues.

Hence, retinas were rinsed in PBS and incubated with primary RNA-Binding Protein with multiple splicing (RBPMS) antibodies (Phospho Solutions, Aurora, CO, USA, 1832-RBPMS, 1:500) diluted in PBS containing 2% *v*/*v* Triton X-100 and 5% *v*/*v* Fetal Bovine serum for 48 h at 4 °C. Subsequently, retinas were washed in PBS and incubated with secondary anti-guinea pig antibodies with alexa fluor 633 nm (Thermo Fisher Scientific, Waltham, MA, USA; A-21105, 1:200) for 48 h at 4 °C. Together with secondary antibodies, Mitotracker Orange (Thermo Fisher Scientific, Waltham, MA, USA; M-7511; dilution 1:200) was also diluted in the staining solution in order to analyze the mitochondrial intensity. At the end, retinas were rinsed in PBS and flat mounted on polarized glass slides and cover slipped with a mounting medium containing DAPI (Vector Laboratories, Inc., Burlingame CA, USA; H-1500) for the cell nuclear staining.

### 2.4. Immunofluorescence Analysis and Quantification for RGC and Mitochondria

Flat-mounted retinas were scanned using a Leica TCS SP5 confocal microscope (Leica Microsystems Inc., Buffalo Grove, IL, USA) in order to acquire a total z-stack thickness of about 90 µm with a sampling thickness of 1 µm, including the inner retina layers. Each retina was sampled at each eccentricity (0.5, 1.5 mm from the center of the optic nerve), and RBPMS positive cells were counted in 8 sampling fields (0.25 × 0.25 mm each) in a masked manner. All the images were processed using the licensed software Leica LAS-X to obtain z-stack maximum projections and multichannel images.

RGC densities were calculated by dividing the number of RBPMS positive cells by the analyzed area. The mean RGC soma size was calculated by dividing the total area covered by the RBPMS positive cells by the number RBPMS positive cells in the same area. The mitochondrial density within RGCs was calculated by the intensity of integrated of mitotracker staining colocalized within RBPMS positive cells.

All the data showing immunostaining quantifications were the average the of the eight samples of the same retina. Nine retinas were analyzed (3-month-old, *n* = 3; 13-month-old control, *n* = 3; 13-month-old NAM-treated, *n* = 3).

### 2.5. Statistical Analysis

Data were analyzed by the Shapiro–Wilk test to verify their normal distribution. Relevant data were graphically displayed and statistically analyzed with JMP Pro 14.2 (SAS Institute Inc., Cary, NC, USA; SPSS (IBM SPSS V26) using repeated measure ANOVA, GEE, and one-way ANOVA followed by Neumann–Keuls post-test. Data are expressed as means ± SEM of the reported *n* values.

## 3. Results

### 3.1. NAM Supplementation Rescues RGC Function and Adaptation Dynamics in D2 Mice

**Figure 1:**
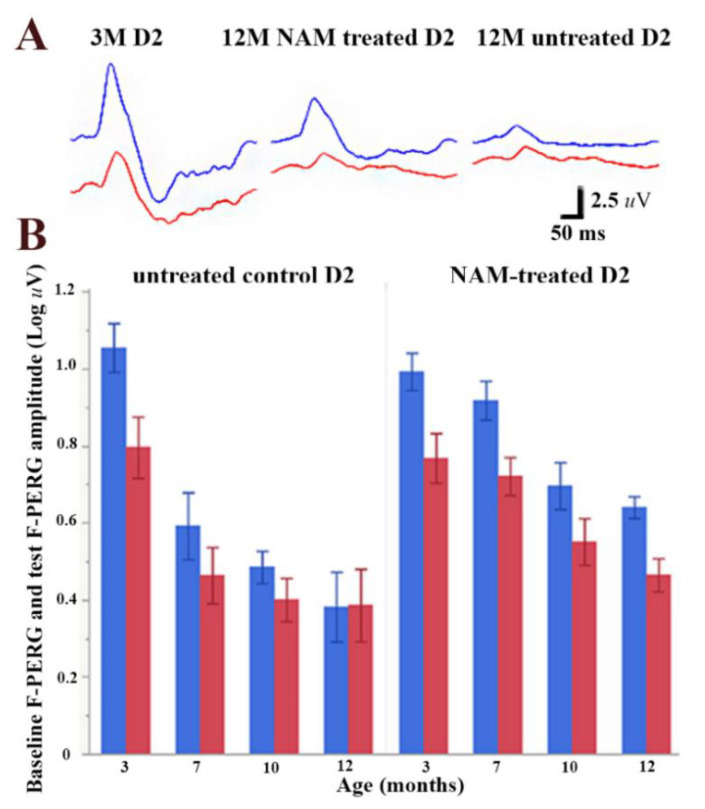
Retinal ganglion cell (RGC) function measurements by pattern electroretinogram (PERG) superimposed with flicker light at 101 or 11 Hz. (**A**) Examples of baseline (blue) and test (red) PERG waveforms recorded D2 mouse at 3 months of age and at 12 months of age with either standard diet or vitamin B3-enriched diet. Note that B3-enriched diet has a protective effect of PERG amplitude; (**B**) Adaptation of F-PERG in D2 mice as a function of age. The amplitude difference between baseline F-PERG and test F-PERG diminished with age in the control group but not in B3-treated group (*n* = 10 for each group). Error bars represent the SEM.

Figure 1A shows representative waveforms of baseline F-PERG (PERG with superimposed 101 Hz flicker, in blue) and test F-PERG (PERG with superimposed 11 Hz flicker, in red) of 3-month-old D2 mice; baseline and test F-PERG of 12-month-old untreated D2 mice; baseline and test F-PERG of 12-month-old D2 mice fed daily with NAM-enriched diet supplement. Note that the baseline F-PERG of the 12-month-old untreated mouse is much reduced compared to the baseline F-PERG of the untreated 3-month-old mouse, whereas the F-PERG amplitude of the NAM-treated mouse is about twice that of the untreated 12-month-old mouse. Figure 1B shows age-related changes of mean baseline F-PERG amplitude (blue bars) together with corresponding mean amplitudes of test F-PERG (PERG with superimposed 11 Hz flicker: red bars). Note that the baseline F-PERG and the test F-ERG decrease with increasing age (RM-ANOVA, *p* < 0.0001) in both untreated and NAM-treated mice. However, for both baseline F-PERG and test F-PERG, the amplitude decline with age was substantially shallower in an NAM-treated group compared to the control group (interaction between baseline F-PERG amplitudes and treatment, *p* = 0.0135). At the endpoint, the difference between baseline and test F-PERG (F-PERG adaptation) was abolished in control D2 mice while preserved in NAM-treated D2 mice (interaction between F-PERG adaptation and age, *p* = 0.0165). We considered a possible role of IOP in the difference of flicker-induced PERG adaptation between control and NAM-treated B2 mice. The average IOP over the observation period was 19.0 mmHg (SE 0.98) in untreated D2 and 17.7 mmHg (SE 0.83) NAM-treated D2 (*p* = 0.29), in agreement with a previous report that NAM-treatment does alter IOP in D2 mice [10].

### 3.2. NAM Supplementation Rescues RGCs and Mitochondria in D2 Mice

**Figure 2:**
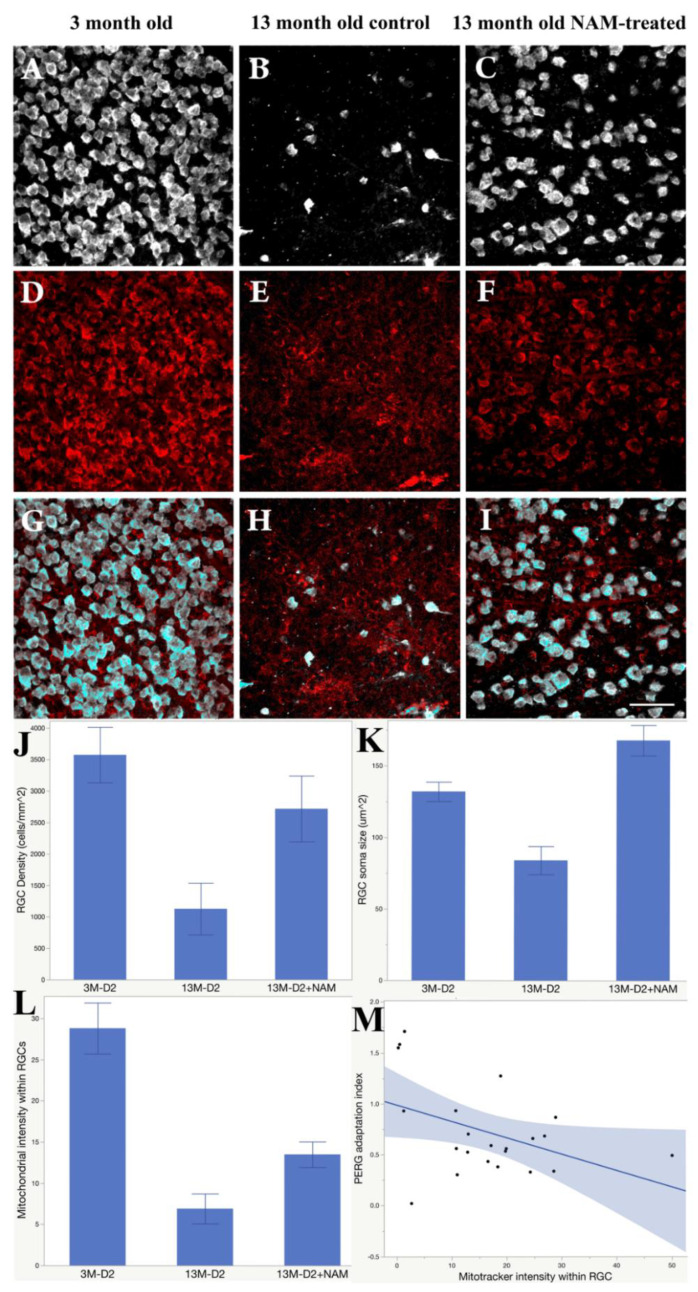
NAM supplementation rescues retinal ganglion cell (RGC) and mitochondria, and preserves flicker-induced PERG adaptation. Examples of RBPMS-labeled RGCs (**A**), mitotracker (**D**), and merged labeling (**G**) in 3-month-old D2 mice. (**B**,**E**,**H**) corresponding examples for 13-month-old control mice. (**C**,**F**,**I**) corresponding examples for 13-month-old NAM-tread mice (Scale bar: 50 µm). (**J**) Mean (SE) RGC density (cells/mm^2^); (**K**) mean (SE) RGC soma size (µm^2^); (**L**) mean (SE) mitotracker intensity for the three groups of mice. (**M**) The Flicker-PERG adaptation index (test/baseline) decreases with increasing mitotracker intensity.

Figure 2 summarizes the effect of NAM supplementation on RGC densities, RGC soma sizes, and the mitochondria intensities within RGCs, together with representative examples of RBPMs (Figure 2A–C) and mitotracker staining (Figure 2D–F). In 13-month-old D2 control retinas (Figure 2E), mitochondrial staining displayed a punctate pattern and was not evenly distributed in the cell soma compared with either the 3-months-old control (Figure 2D) or the 13 months-old treated retinas (Figure 2F) (*n* = 3 retinas per each group). The intensities of mitochondria staining in NAM treated retinas (Figure 2F) are 3.75 times higher than in untreated retinas (Figure 2E). The mitotracker staining within RGC displayed a drastic decrease in the intensity of active mitochondria in 13-month-old age control retinas (Figure 2H) compared with nicotinamide treated retinas (Figure 2I). The bar graph (Figure 2J) shows the RGC densities were significantly (*p* < 0.01) preserved in the nicotinamide treated D2 retinas, and there is severe loss of RGC in age control D2 retinas at 13 months old. RGC soma size (Figure 2K) in the nicotinamide treated retinas in 13-month-olds have no difference with RGC soma size in 3-month-old D2. RGC soma size has been significantly reduced half (*p* < 0.01) in D2 age control compared with the treated group at 13 months old. Mitochondrial within RGC intensities are significant difference (*p* < 0.01) between untreated age control and nicotinamide treated retina (Figure 2L). The nicotinamide treatment restored 2 times of mitochondria intensities within RGCs in 13-month-old D2 treated retinas compared with the untreated age control. Figure 2M shows the association between F-PERG adaptation and mitochondrial intensities within RGCs. The index of F-PERG adaptation was defined as a ratio between test F-PERG amplitude and baseline F-PERG amplitude. When there is a F-PERG adaptation, the index is <1. Figure 2M shows that the stronger F-PERG adaptation, the higher the mitochondrial intensity inside the RGCs (*p* = 0.0464).

## 4. Discussion

A number of studies have investigated in different mammals the magnitude and temporal dynamics of flicker-induced changes of retinal arterial diameter and blood flow [16,23], which is caused by increased metabolic demand of inner retinal neurons. We developed a tool to assess flicker-induced adaptation of RGC function as measured by PERG with superimposed flicker (F-PERG) [18]. The F-PERG has been used in this study to test whether RGCs’ ability to adapt their function upon flicker challenge is reduced in the D2 mouse model of glaucoma, and whether it is preserved in D2 mice fed with nicotinamide-rich diet that supports mitochondrial function. Our results show that the F-PERG adaptation was progressively reduced with increasing age in D2 mice fed with standard diet but was preserved in D2 mice fed with nicotinamide-diet. At 12 months of age, when F-PERG adaptation was abolished in control mice, F-PERG adaptation of nicotinamide-fed D2 mice was similar to that of 3-month-old control mice. The effect was not due to IOP-related changes, in agreement with previous results [10]. At 13 months of age, RGC density, RGC soma size, and mitotracker staining were dramatically reduced in control D2 mice but were largely preserved in nicotinamide-fed D2 mice. In addition, the magnitude of F-PERG adaptation was inversely proportional to the intensity of mitotracker staining. The present results complement previous reports that PERG and RGC death are spared in nicotinamide-fed D2 mice [10]. NAM supplementation is believed to decrease the probability of metabolic and/or energetic failure, which is age-related and prominent in D2 mice [10]. Altogether, the present results indicate that, in the D2 model of glaucoma, RGCs progressively lose the ability to autoregulate their function in response to a flicker-induced metabolic challenge, whereas the flicker-induced RGC functional autoregulatory ability is preserved in nicotinamide-fed D2 mice, in association with reduced RGC death and reduced mitochondria depletion.

## 5. Conclusions

Flicker-induced PERG changes (F-PERG adaptation) provide a means to investigate the autoregulatory dynamics of RGC function in response to temporarily increased metabolic demand. F-PERG adaptation is disrupted in aged D2 mice but is preserved in D2 mice fed with a NAM-enriched diet, which supports mitochondrial function. F-PERG adaptation may be a promising non-invasive tool to probe early changes of RGC autoregulatory dynamics in optic nerve diseases, in order to assess the effect of dietary/pharmacological treatments and help predict the fate of RGCs with or without treatments.
